# The user-centered design and development of a childhood and adolescent obesity Electronic Health Record tool, a mixed-methods study

**DOI:** 10.3389/fdgth.2024.1396085

**Published:** 2024-09-18

**Authors:** K. Taylor Bosworth, Parijat Ghosh, Lauren Flowers, Rachel Proffitt, Richelle J. Koopman, Aneesh K. Tosh, Gwen Wilson, Amy S. Braddock

**Affiliations:** ^1^ Department of Family and Community Medicine, School of Medicine, University of Missouri, Columbia, MO, United States; ^2^ School of Medicine, Tom and Anne Smith MD/PhD Program, University of Missouri, Columbia, MO, United States; ^3^ School of Medicine, University of Missouri, Columbia, MO, United States; ^4^ School of Health Professions, University of Missouri, Columbia, MO, United States; ^5^ Department of Child Health, School of Medicine, University of Missouri, Columbia, MO, United States

**Keywords:** Electronic Health Record (EHR), graphic design, UI, user-centered design, shared-decision making

## Abstract

**Background:**

Childhood and adolescent obesity are persistent public health issues in the United States. Childhood obesity Electronic Health Record (EHR) tools strengthen provider-patient relationships and improve outcomes, but there are currently limited EHR tools that are linked to adolescent mHealth apps. This study is part of a larger study entitled, CommitFit, which features both an adolescent-targeted mobile health application (mHealth app) and an ambulatory EHR tool. The CommitFit mHealth app was designed to be paired with the CommitFit EHR tool for integration into clinical spaces for shared decision-making with patients and clinicians.

**Objectives:**

The objective of this sub-study was to identify the functional and design needs and preferences of healthcare clinicians and professionals for the development of the CommitFit EHR tool, specifically as it relates to childhood and adolescent obesity management.

**Methods:**

We utilized a user-centered design process with a mixed-method approach. Focus groups were used to assess current in-clinic practices, deficits, and general beliefs and preferences regarding the management of childhood and adolescent obesity. A pre- and post-focus group survey helped assess the perception of the design and functionality of the CommitFit EHR tool and other obesity clinic needs. Iterative design development of the CommitFit EHR tool occurred throughout the process.

**Results:**

A total of 12 healthcare providers participated throughout the three focus group sessions. Two themes emerged regarding EHR design: (1) Functional Needs, including Enhancing Clinical Practices and Workflow, and (2) Visualization, including Colors and Graphs. Responses from the surveys (*n* = 52) further reflect the need for *Functionality* and *User-Interface Design* by clinicians. Clinicians want the CommitFit EHR tool to enhance in-clinic adolescent lifestyle counseling, be easy to use, and presentable to adolescent patients and their caregivers. Additionally, we found that clinicians preferred colors and graphs that improved readability and usability. During each step of feedback from focus group sessions and the survey, the design of the CommitFit EHR tool was updated and co-developed by clinicians in an iterative user-centered design process.

**Conclusion:**

More research is needed to explore clinician actual user analytics for the CommitFit EHR tool to evaluate real-time workflow, design, and function needs. The effectiveness of the CommitFit mHealth and EHR tool as a weight management intervention needs to be evaluated in the future.

## 1 Introduction

Childhood and adolescent obesity are prevalent and lasting public health issues, with 20.7% of children (aged 6–11 years old) and 22.2% of adolescents (aged 12–19 years old) having obesity in the United States from 2017 to 2020 (1). Childhood and adolescent obesity can impact health in several ways: obesity-related high blood pressure, high cholesterol, type 2 diabetes, asthma, sleep apnea, and joint problems (1). Additionally, around 80% of those with obesity during adolescence are likely to have obesity as an adult (2, 3). Due to the continuing rise in childhood and adolescent obesity (4), there is a substantial need for novel interventions that improve weight and lifestyle management (5–7). As a result, we developed the CommitFit mobile health (mHealth) app for adolescents (8). Further, we designed the CommitFit Electronic Health Record (EHR) tool to empower healthcare providers to leverage detailed user-reported data extracted from the mHealth app. This may enhance shared decision-making processes, thereby optimizing patient care outcomes.

Shared decision-making describes the process of patients and clinicians working together to create optimal treatment plans in the context of health care. As a result, patients are encouraged to be more engaged and interactive with the care they choose to receive. Shared decision-making interventions have demonstrated several benefits: they enhance patients’ understanding of treatment options, increase the number of patients with realistic expectations about benefits and risks, encourage patient participation in decision-making, and align treatment choices with patients’ values (9, 10). Additionally, integrating patient preferences into the decision-making process can improve patient well-being by fostering better treatment adherence, reducing concerns about illness, and increasing satisfaction with health outcomes (9–12), especially in obesity-related care and management (13–15). Shared decision-making may also be appropriate for adolescent and child populations, but it is not always leveraged and has its own set of challenges (16–18).

Tools like the CommitFit mHealth app and EHR tool can facilitate shared decision-making among clinicians, caregivers, and adolescent patients. However, to be truly effective, these tools must have a user interface designed with the users’ preferences and needs in mind. Therefore, a user-centered design process is essential to ensure the tool meets user requirements (19, 20). The user-centered design process (21, 22) described in this study for the CommitFit EHR tool involves four main steps: (1) Early and interactive involvement of users throughout the design process, (2) Orient users to focus on clinically-relevant tasks and user requirements, (3) Incorporation of feedback into the tool's development, and (4) Iterative design improvements through prototyping.

While various approaches to managing childhood and adolescent obesity have been employed, including educational efforts targeting parents and youths (23) and medical or surgical interventions, the public health crisis persists. Educational initiatives aim to promote healthier lifestyles but often struggle to produce sustained behavioral changes among individuals (24). Medical and surgical interventions can be effective for individual patients but may not be accessible or scalable solutions for the broader population or public health (25). This is especially true with the rise of glucagon-like peptide 1 (GLP-1) agonists for weight loss, while effective (26), are often expensive (27) and inaccessible to many. Technology-driven approaches, despite their innovative potential, frequently fail to translate into practical or actionable insights for clinicians. Strategies such as evaluating digital addiction (28) and utilizing machine learning models to classify and predict obesity levels (29) have been explored. However, these methods have limitations in their ability to meaningfully influence clinical practice.

To address this gap, tools like the CommitFit mHealth app (8, 30) and CommitFit EHR tool (31) are designed to integrate into clinical workflows and facilitate shared decision-making among clinicians, caregivers, and adolescent patients. By focusing on user-centered design and ensuring that these tools meet the specific needs of users, we can enhance their effectiveness in clinical practice and make a more meaningful impact on the management of childhood and adolescent obesity.

### 1.1 Previous work: Commitfit mHealth app

**Figure 1:**
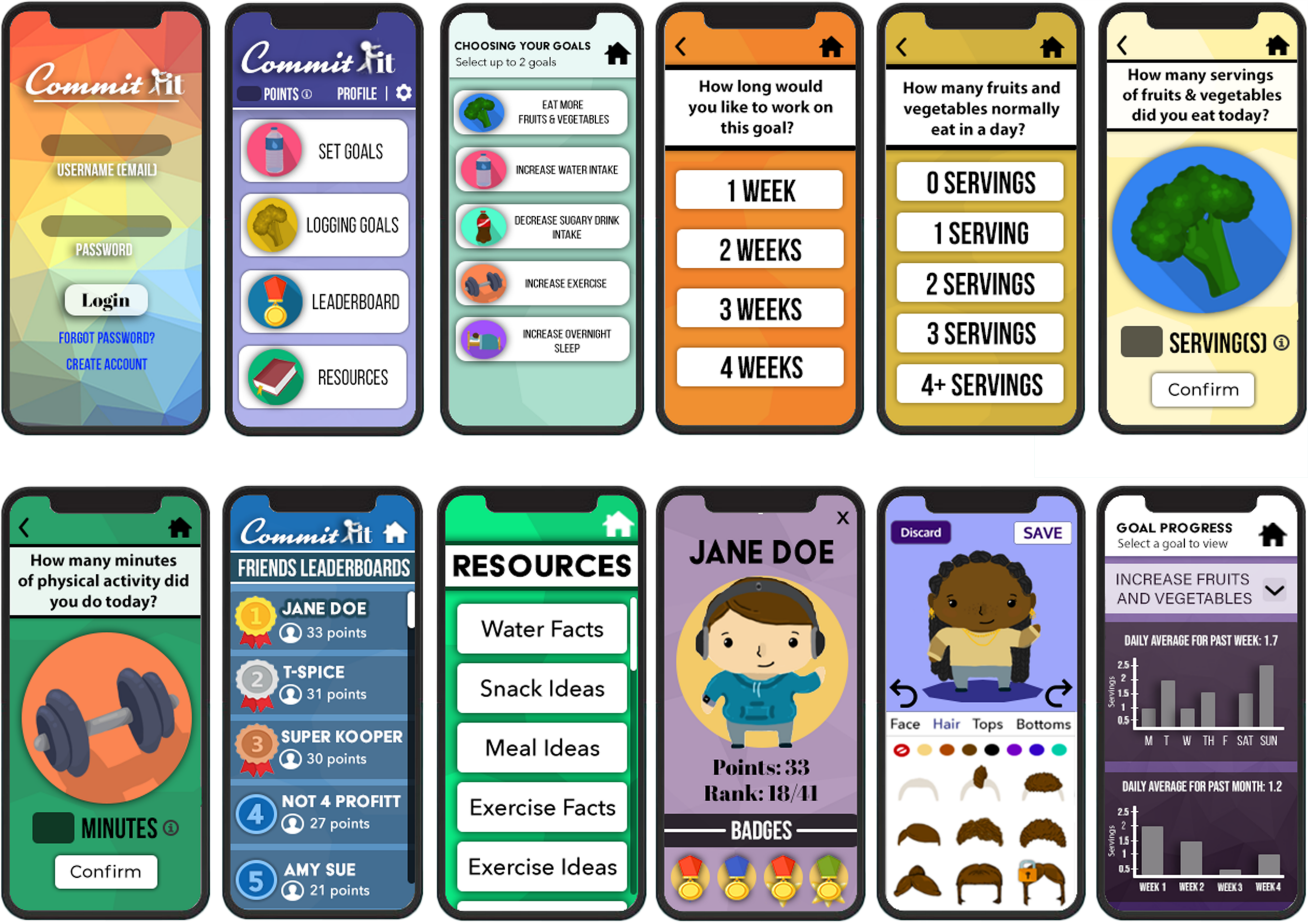
Sample screenshots of the CommitFit mHealth app designed for adolescents. The user-reported data from adolescent patients using the CommitFit mHealth app is planned to be integrated with the paired EHR tool. The top row of wireframes (from left to right) includes screens for login, dashboard, goal selection, time commitment to the goal, baseline amount, and a summary with goal confirmation. The bottom row of wireframes (from left to right) features screens for daily health behavior logging, leaderboard, resources, profile, gear store, and goal progress.

This study is part of a larger study entitled, CommitFit, which features both an adolescent-targeted mHealth app, the “CommitFit mHealth app”, and an ambulatory EHR tool, the “CommitFit EHR tool”. The CommitFit mHealth app was designed to be paired with the CommitFit EHR tool for integration into clinical spaces for shared decision-making with patients and clinicians. The CommitFit mHealth app is targeted at adolescent patients and consumers (8, 30). The bottom-up design of the CommitFit mHealth app was informed by relevant collaborators: adult caregivers, adolescents, health professionals, and technologists (such as programmers and designers). In the app, adolescent users are incentivized through virtual points and coins collected in the app to enter health behavior data daily. The basic function of the CommitFit mHealth app is to allow adolescent users to select a maximum of two of five health behavior goals including: (1) increase physical activity, (2) increase the intake of fruits and vegetables, (3) decrease the consumption of sugar-sweetened beverages, (4) increase water consumption, and (5) improve overnight sleep. After behavior selection, users choose a daily goal level based on their current health behavior, for example, a user who currently consumes no fruits/vegetables may set a goal of two servings per day. Users will use the “logging goals” functionality of the app to monitor their daily behaviors and be prompted to log their goals daily with a customizable reminder. Users are then ranked individually or on a team-based leaderboard based on the points they accrue from logging and achieving their health behavior goals. They can also use points to purchase clothes and gear in the app. Below is a sample of the CommitFit mHealth app (Figure 1).

The conception of the CommitFit mHealth app is based on enhancing current in-clinic practices, while actively engaging adolescents to lead their own health behavior changes. The CommitFit mHealth app, and its resulting user-entered data, are designed to be integrated into the CommitFit EHR tool. The CommitFit EHR tool was designed with the integration of the data collected by the CommitFit mHealth app in mind. The CommitFit EHR tool is intended to be a health provider-facing EHR tool (8). The CommitFit EHR tool can be utilized in clinics to facilitate shared decision-making about the user's health behaviors and health metrics.

### 1.2 Knowledge gap and rationale

Recent studies found that childhood obesity EHR tools strengthen provider-patient relationships and improve outcomes (32). Yet, current EHR tools for adolescent mHealth apps are limited in number and capacity (8, 33, 34). EHR tools may enhance provider satisfaction; however, poor usability of EHRs has been linked to patient safety issues. Poor usability in design has been characterized by small buttons, excessive clicks, and inefficient or complex layouts (35). Despite the known risks to patient safety, poorly designed EHR tools remain common due to the need to represent vast, complex, and diverse information (36, 37). EHR developers must assess provider preferences prior to implementation. The presentation of consistent and accurate health behavior data can support clinical and shared decision-making for health providers and professionals partnering with patients.

### 1.3 Objectives

This mixed-methods investigation aims to use both qualitative and quantitative data analyses to identify the essential functional and design requirements for developing the CommitFit EHR tool. The focus is on the preferences and needs of healthcare clinicians and professionals for a tool intended for managing childhood and adolescent obesity. By analyzing data from focus groups and surveys, we aim to create a comprehensive understanding of current practices and design needs for an effective EHR pediatric weight management tool.

## 2 Methods

User-centered design is an iterative process that includes the users in each stage of the design process (8, 20, 21, 38). The integration of meaningful stakeholder contributions enhances system designs and various systems or electronic tools by ensuring an intuitive user interface (8, 39, 40). Our user-centered design process was initiated utilizing feedback received in provider focus groups and surveys for development of the EHR data tool.

A mixed-method approach allowed descriptions and understanding of design and the user needs and expectations of providers when employing EHR tools. Due to the iterative nature of our design process, we used rapid qualitative analysis of focus group feedback and quantitative survey results to provide. Rapid qualitative analysis is employed when quick results are needed to modify, refine, or develop various tools (41–43). This type of analysis typically follows an inductive approach (41), as was done in this study. Later, inductive thematic analysis was conducted to provide better contextualization for the qualitative findings.

### 2.1 Focus groups

Physicians and providers were recruited to participate in up to three virtual semi-structured focus groups aimed at refining the design of the CommitFit EHR tool static “paper” prototypes, ensuring the prototype was optimized before programming. Physicians and providers were recruited and enrolled for the focus groups via word of mouth and recruitment emails sent to all MU family medicine and pediatrics faculty members. Eligibility and inclusion criteria included: being a practicing provider (attending physician or PA, NP, etc.) who provides pediatric weight management, English fluency, and being proficient with EHR tools. Candidates were excluded if they were not regular users of EHR tools or health professionals still in training (I.e., medical students, residents, etc.). The study plan was approved by the University of Missouri Health Science IRB (MU #2054598). Informed consent was obtained from all participants before partaking in the study.

All participants received compensation in the form of a $50 e-gift card of their choice to one of three popular vendors, for participating. The providers were recruited from various demographic backgrounds including race, gender, and years in practice.

Focus group sessions lasted one hour each and were conducted virtually on Zoom©. Participants were debriefed on the CommitFit mHealth app and its planned integration into the EHR tool before viewing the EHR tool; this debriefing session included a brief, less than 10-minute, walk-through of the mHealth app and its features. We then presented the prototype which stimulated the flow of the CommitFit EHR tool to the participants to receive specific feedback. Participants were asked open-ended questions using a semi-scripted guide, as shown in Table 1.

**Table 1:** Sample of open-ended questions asked to focus group participants.

Visual design	Usage	Features and functionality	Current in-clinic needs and practices
What can we do to improve the design, format, or data displayed in the tool to make it more useful?	How do you see CommitFit fitting into your workflow? Would you use the EHR visualization tool to discuss this information with your patients?	What other elements could an EHR tool have that you think would be helpful?	Do you feel that you currently have effective tools for obesity management? How about adolescent obesity?
Is there anything else you want us to consider when designing the CommitFit EHR tool, like how or when you would use it and ways to make it easier to use?	How do you think having CommitFit health behavior data would influence the conversation between you and your patients about setting health behavior goals?	Do trends matter to you in how you treat adolescent obesity? What data would you like to see on a line graph? Would you prefer to see them together on the same graph or separate?	Tell me about your current workflow in visits where you counsel adolescents about weight, either in visit specifically for child obesity or in annual well child visits?
	How much of a difference would these conversations have with your patients? Would it change how you approach in-clinic counseling?	What basic health behavior elements would you want CommitFit to have? Which behaviors would you recommend to adolescents with obesity?	When treating adolescent obesity, what are the most important data that you would like to see in an EHR visualization?

These questions are placed within four categories: Visual Design, Usage, Features and Functionality, and Current In-Clinic Needs and Practices.

**Table 2:** Contents of the 38-item survey administered to participants.

(1) Demographics	(2) Current Practices	(3) Preferences	(4) CommitFit EHR tool
*Select the most applicable option*	*Rate these statements from 0 (strongly disagree) to 100 (strongly agree)*.	*Choose all that apply*	*Please answer the questions below based on how you see yourself using this EHR tool with patients in clinic*. *Rate these statements from 0 (strongly disagree) to 100 (strongly agree).*
1.1: Which degree have you completed? • MD • DO • DNP • PA • MSN (CNS, NP, etc)	2.1: I have effective child/adolescent obesity management tools in-clinic.	3.1: What health information would you like to see in an EHR BMI percentile over time tool to help address obesity in adolescent patients? • Blood Pressure percentile over time • Current weight in kilograms • Current weight in pounds (lb) • Change in weight since last clinic visit • Change in BMI percentile since last clinic visit • Systolic and diastolic blood pressure • Blood Pressure percentile • Most recent HbA1C • Most recent lipid panel: cholesterol, LDL, HDL, triglycerides • Most recent glucose Most recent liver function tests • Growth Charts • Other (specify below)	4.1: I like the layout of this tool.
1.2: If you are a physician, are you a(n): Attending *Note: Midlevel providers, please select “attending” option* • Attending • Resident^a^	2.2: I effectively provide healthy behavior lifestyle counseling for patients during Well Child visits.	3.1.a: Other health information: _________	4.2: I like the colors of this tool.
1.3: What is your specialty? • Family Medicine • Pediatrics • Other	2.3: I effectively provide healthy behavior lifestyle counseling for patients during child obesity visits.	3.2: Which do you prefer when visualizing lifestyle data (i.e., fruits and vegetables or water intake) logged by your pediatric/adolescent patient? • Line graphs • Bar graphs	4.3: I like the look and feel of this tool.
1.4: Subspecialty field, if applicable: _______	2.4: Pediatric/adolescent patients follow my health behavior recommendations.	3.3: Which do you prefer when visualizing lifestyle data (i.e., fruits and vegetables or water intake) logged by your pediatric/adolescent patient? • Monthly Averages • Weekly Average	4.4: I anticipate using this tool in the clinic.
1.5: How many years have you been practicing? • <1 year • 1–3 years • 3–5 years • 5–10 years • >10 years	2.5: The current health care system provides sufficient resources for my pediatric/adolescent patients to make meaningful health behavior changes.	3.4: Which do you prefer in a graph? • Combined weight and BP (line graph) • Separate weight (or other biometric) in line graph	4.5: I would recommend using this tool to others.
1.6: Please select your gender identity. • Cis-man • Cis-woman • Non-binary • Transman • Transwoman • Prefer not to answer • Other	2.6: The current health care system provides sufficient continuity for my pediatric/adolescent patients to make meaningful health behavior changes.	3.5: How would you want patient logged lifestyle data to flow into your clinic note? • As an autotext with an average over the past 4 weeks • An autotext with an average over the past 4 months • As an autotext with an average over the entire time period they have been logging the goal • Average with the minimum and maximum range • An option to copy and paste averages from the EHR visualization • Patient self-reported health behavior goals • Minimum nutrition and physical activity documentation requirements for well child visits • Other (specify below)	4.6: Information from this tool will help me provide shared decision making with my patients.
1.7: Please select all applicable to your race. • Black or African American • American Indian or Alaska Native • Asian • Native Hawaiian or Other Pacific Islander • White • Some other race	2.7: I have sufficient training to provide healthy behavior lifestyle counseling for pediatric/adolescent patients.	3.5.a: Other: _________	4.7: I can retrieve the information I need easily using this tool.
1.8: Please select your ethnicity. • Hispanic or Latino • Not Hispanic or Latino	2.8: I have sufficient training to provide obesity management for pediatric/adolescent patients.		4.8: I anticipate that this tool will be useful when I provide health behavior counseling in my clinic.
1.9: What is your age (years)? • <20 • 20–30 • 31–40 • 41–50 • 51–60 • >60 • Decline to answer	2.9: Current EHR data supports health behavior lifestyle conversations with adolescents/children.		4.9: I anticipate that this tool will make providing health behavior counseling in my clinic easier.
1.10: Do you treat children/adolescent patients? • Yes • No^a^	2.10: I see a need for tools like mobile health (mHealth) apps to help patients to develop healthy lifestyle habits.		
1.11: Do you treat obesity in your clinic? • Yes • No^a^			
1.12: Do you treat childhood/adolescent obesity in your clinic? • Yes • No			

This survey consisted of four main sections: (1) Demographics, (2) Current Practices, (3) Preferences, and (4) CommitFit EHR Tool.

^a^ Indicates that, if option is selected, survey ends and does not capture the survey data.

Each virtual focus group session was video recorded. The recordings of the focus group sessions were utilized to produce verbatim transcripts using Microsoft 365. Researchers reviewed the video for transcription errors. Identifying information, such as the names of participants, has been removed from the qualitative quotes to ensure confidentiality. Transcripts were used by the research team in qualitative analysis of focus group feedback.

Rapid qualitative analysis was done after each focus group session to provide iterative design development of the CommitFit EHR tool, which quickly identified areas of improvement (43). The rapid qualitative analysis was integrated into the user-centered design process (20, 44), with focus groups providing early and active user involvement. For the rapid qualitative analysis, transcripts were evaluated for feedback regarding design elements (such as color and layout) and functionality aspects (such as graph displays). Once evaluated, the proposed changes to the CommitFit EHR tool were presented to the research team to confirm that changes were in line with participant feedback. We explored these elements though questions regarding *Visual Design, Usage, Features and Functionality,* and *Current In-Clinic Needs and Practices* (see Table 1), with this approach being determined *a priori*. This approach allowed us to identify and investigate user and task requirements. We prioritized design decisions that affected clinical workflow and shared decision-making, as indicated by participants. User feedback from the rapid qualitative analysis was directly used to implement changes to the CommitFit EHR tool. These changes were made iteratively between each focus group session, with sessions 2 and 3 used to confirm that the modifications we made were aligned with participants’ expectations.

Later, inductive qualitative thematic analysis was performed on these transcripts using Dedoose Version 9.0. This was done to contextualize and determine major themes for the clinical (31) and design needs via codes (45). The qualitative thematic analysis was performed by two independent coders (PG, ASB) until consensus was met. Another researcher (KTB) acted as a third independent coder, if a coding agreement was not met.

### 2.2 Surveys

Physicians and providers were recruited to participate in a 38-item survey (see Table 2) to inform the final design (Version 4) of the CommitFit EHR static prototypes, to ensure the best iteration of the EHR tool before programming. Eligibility and inclusion criteria included: being a practicing provider (attending physician or practicing clinician (PA, NP), managing children or adolescents with obesity in their primary care or specialty clinic, and utilizing EHR tools. Candidates were excluded if they had not previously treated child or adolescent obesity or were health professionals still in training (I.e., medical students, residents, etc.). An informed consent document was disclosed to all participants in the online survey before the study. Participants screened themselves for the inclusion and exclusion criteria.

**Figure 2:**
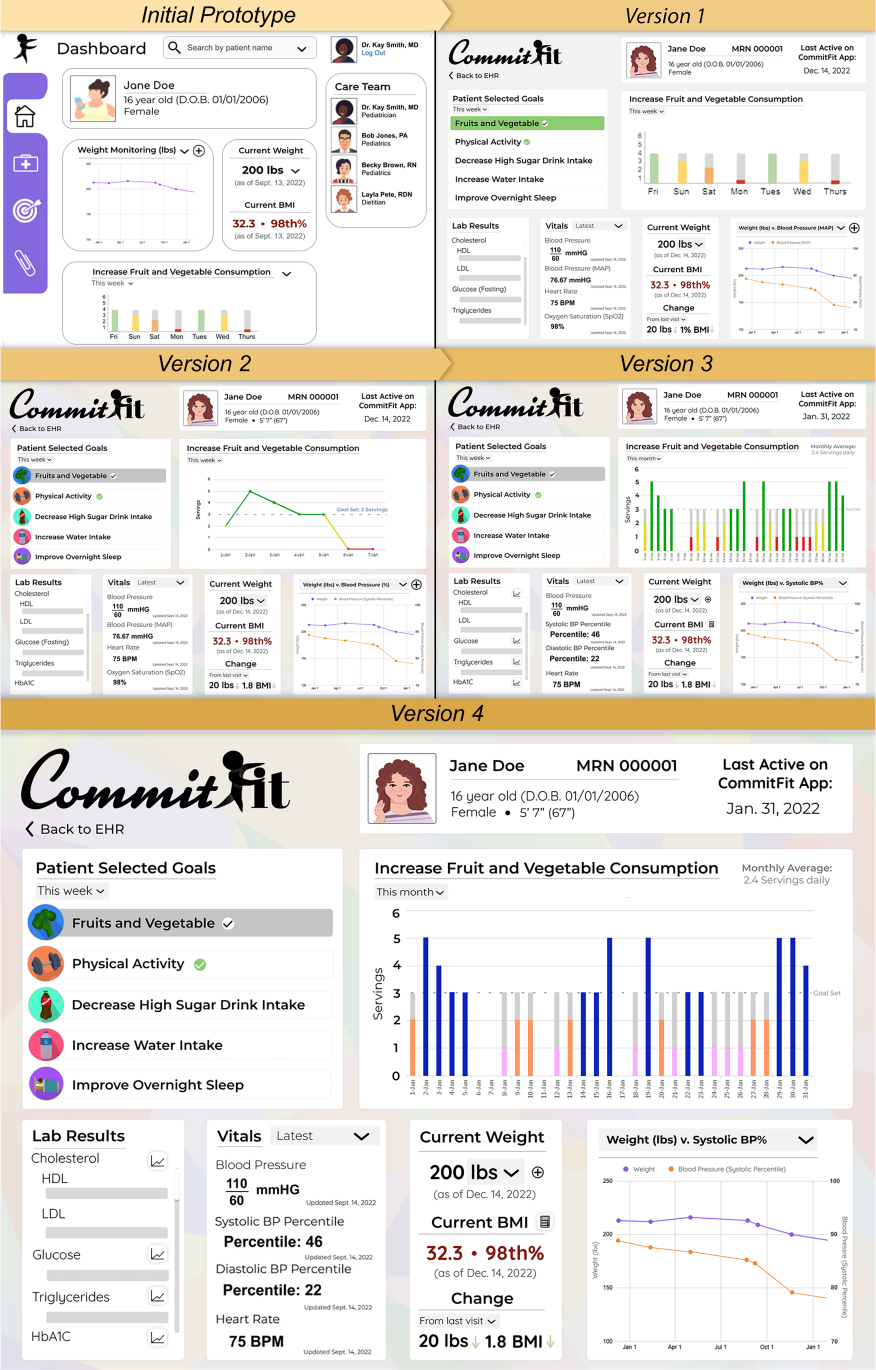
Overview of the evolution of EHR iterative designs. Versions 1, 2, and 3 were shown to Focus Group sessions 1, 2, and 3, respectively. Version 1 was updated for a more modern design. Version 2 reflected improved colors, added buttons to display trends for Lab Tests, and implemented line graph featuring a “Goal Set” indication. In Version 3, the main “Patient Selected Goal” line graph was converted back to a bar graph and a monthly or weekly average goal amount counter was added. The colors in Version 4 were updated to remove the “traffic light” color scheme. Version 4 was developed using feedback from the surveys and Focus Group session 3.

The survey was administered utilizing Qualtrics© (for University of Missouri faculty only) and REDCap© (for remaining participants). The transition was made to allow for social security numbers to be collected for compensation in REDCap, which were not required for University of Missouri faculty and employees. The survey consisted of four sections: *(1) Demographics, (2) Current Practices regarding child and adolescent obesity management, (3) Preferences for EHR tools,* and *(4) questions regarding the specific CommitFit EHR Tool.* Section *(4) CommitFit EHR Tool* (see Table 2) evaluated Version 3 of the paper prototype (see Figure 2). This article focuses on the functional and design needs for the CommitFit EHR tool. For the scales, participants rated statements from a numerical scale of 0 (strongly disagree) to 100 (strongly agree), to improve sensitivity (46). Surveys were distributed to assess current in-clinic practices, deficits, and general beliefs and preferences. Additionally, the survey was used to assess additional information regarding the perception of the design and functionality of the CommitFit EHR tool. This survey was chosen over the industry standard System Usability Scale (SUS) to assess specific preferences and opinions regarding the CommitFit EHR tool, particularly focusing on aspects relevant to clinical practices that SUS does not effectively capture.

All participants received the option to receive compensation in the form of a $10 e-gift card of their choice to three popular vendors, for participating. The providers were recruited from varied demographic backgrounds including race, gender, and years in practice.

### 2.3 Commitfit EHR tool and iterative design

Electronic wireframes were adapted into static prototypes and assets using Adobe Photoshop®. Prototypes were presented to participants using Microsoft PowerPoint® to simulate EHR workflow. The CommitFit EHR design was evaluated in a stepwise fashion, with feedback being integrated after each of the three focus group sessions. Feedback from the focus groups was initially utilized using the rapid qualitative analysis, as previously described above. Elements, such as colors, layout, icons, and graphs, were adapted to meet the communicated expectations of users. The evolution of this design from the rapid qualitative analysis is reflected in Figure 2. Each version and design were evaluated by participants in focus groups with the final version 4 developed based on results from the Focus Group session 3 and surveys (see Figure 2).

## 3 Results

### 3.1 Qualitative

A total of 12 healthcare providers participated throughout the three focus group sessions. Five (41.67%) participants self-identify as a cis-man and seven (58.33%) self-identify as a cis-woman. Twelve (100%) participants self-identify as non-Hispanic or Latino White, with one (8.33%) participant identifying as White and American Indian or Alaska Native. Most (*n* = 9, 75.00%) of the participants were MDs, followed by DOs (*n* = 2, 16.67%), and one was a health psychologist in a pediatric weight management clinic (8.33%). Most participants practiced pediatrics (*n* = 7, 58.33%). Four participants (33.33%) practiced Family Medicine. Additionally, our focus group participants represented several subspecialty fields including Pediatric Endocrinology, Clinical Informatics, Developmental Pediatrics, and Health and Clinical Psychology. All participants had over 5 years in practice, with most (*n* = 9, 75.00%) having greater than 10 years in practice. Full self-identified demographic information is available in Table 3.

**Table 3:** Self-reported demographics of healthcare provider focus groups.

Education	*n* (*n*%)
MD	9 (75.00%)
DO	2 (16.67%)
DNP	0 (0%)
MSN	0 (0%)
PA	0 (0%)
Psychologist	1 (8.33%)
Speciality	
Family medicine	4 (33.33%)
Pediatrics	7 (58.33%)
Other	1 (8.33%)
Subspecialty	
Pediatric endocrinology	1 (8.33%)
Clinical Informatics	1 (8.33%)
Developmental pediatrics	1 (8.33%)
Health and clinical psychology	1 (8.33%)
No subspeciality indicated	8 (66.67%)
Years in practice	
<1 years	0 (0%)
1–3 years	0 (0%)
3–5 years	0 (0%)
5–10 years	3 (25.00%)
>10 years	9 (75.00%)
Gender	
Cis-man	5 (41.67%)
Cis-woman	7 (58.33%)
RACE (select all that apply)	
Black or AA	0 (0%)
American Indian or Alaska Native	1 (8.33%)
Asian	0 (0%)
Native Hawaiian or other PI	0 (0%)
White	12 (100%)
Some other race	0 (0%)
Ethnicity	
Not Hispanic or Latino	12 (100%)
Hispanic or Latino	0 (0%)
Age	
<20	0 (0%)
20–30	0 (0%)
31–40	2 (16.67%)
41–50	6 (50.00%)
51–60	4 (33.33%)
>60	0 (0%)
Decline	0 (0%)

### 3.2 Themes and quotes from focus groups

#### 3.2.1 Functional needs: information and workflow needs

##### 3.2.1.1 Enhance clinical practices

Participants expressed the need for the CommitFit EHR tool to facilitate and enhance clinic lifestyle counseling with adolescent patients. Participants wanted the CommitFit EHR tool to be presentable to adolescent patients and their caregivers to help facilitate engagement.

“I could see myself turning [the CommitFit EHR tool] around and showing it to the family when they're there for a visit, like to discuss results. So, just having it, at least in a way that appears, you know, a parent and child could interact with the [CommitFit EHR tool] when we're in the office.” [FG 1]

“It’s just good. I think you can also then kind of, sort of, speak the language that your patient is seeing, even though I know it’s different and this is a provider facing thing dashboard, it still just allows you to have that same language the same way to talk about stuff. And so anyway, I hope that helps, but I do really like how this looks. It looks really good.” [FG 1]

##### 3.2.1.2 Workflow: notifications and summaries

Participants also wanted summaries of CommitFit App use, enrollment, and data analytics sent to the clinician on the day of the clinic visit. Participants shared ideas of where and how they would like to receive notifications or summaries about CommitFit use.

“.. it would be super awesome to have a summary page that basically helps me see. Uhm, where we're at around this particular health condition.’ [FG 1]

“It would be nice if it keeps track of that in some way that I would get a report like, if you're my patient, [name] that says you know [name]’s logged into the device three times in the last week. Follow up or three months follow up and what are we going to be talking about like you know do we have an idea that they're staying pretty motivated or they're not motivated and so having something that lets me know before the day of the appointment or they actually utilizing the app and just so we'd have that data.” [FG 1]

“I do think maybe a quick notification in the ER [Electronic Record], just like when they go to the emergency room or something like that. Just a one line or nothing detail, just you know, this patient is enrolled in it..” [FG 3]

#### 3.2.2 Data visualization

##### 3.2.2.1 Color

The colors within the CommitFit EHR tool must be impactful and intentional to improve usability. Colors can highlight important elements, which increases usability and improves visualization.

“That you wouldn't want to grey them out because they seem less important, but to highlight what they're focused on. Just like with increasing that little check mark.” [FG 3]

“I think about PowerChart [an EHR product by Cerner®] kind of visualizations. It’s really hard to kind of land your eyes on what it is that you care about, and so this is great ‘cause the colors are helpful.” [FG 1]

“The grey is helpful just for to catch my eye to say OK, those were the days that they didn't. It feels better than just having the red and yellow, so I would keep the grey to the goal line.” [FG 3]

In Prototype Versions 1–3, the evidence-based (36, 47) “traffic light” color scheme was used in the “Patient Selected Goals’ graph (green for good, yellow borderline, red bad); however, participants in Focus Group 3 voiced that this color scheme, particularly red, has negative connotations and implies failure which could frustrate and demotivate patients. These concerns are especially impactful when considering the CommitFit EHR tool could be used to facilitate clinician-patient conversations and may be occasionally patient-facing.

“I mean it depends like if there’s negative connotation about red is bad or red is stop and you're worried about, like, telling kids, you know. Like, if you don't meet your goal, that’s a fail, right? I mean, we calculate red with check marks, so I don't know, you know.” [FG 3]

##### 3.2.2.2 Graphs

Additionally, the readability of graphs was a key component of visualization. During the focus groups participants preferred bar graphs over line graphs for the “Patient Selected Goals” graph:

“I like having just the bar for each day is probably going to make it look a lot nicer, make it simpler, easier to see, and I feel like easier to look at the goals to kind of where it is vs. the goals if you have less, kind of, a busy schematic.” [FG 2]

“Instead of the chart, so you know, instead of these peaks and valleys [line graph in Version 2], just on the date. So, each day is just a square, instead of these graphs.” [FG 2]

### 3.3 Quantitative

Fifty-two complete surveys were analyzed. A majority (*n* = 48, 92.31%) of participants were MDs and the area of practice was split between family medicine and pediatrics (53% vs. 44%). Compared to the focus group sample, survey respondents were more racially diverse, younger, and in practice for fewer years. A total of 28 (53.85%) of the survey participants were Family Medicine providers and 23 (44.23%) were Pediatric providers. One survey participant (1.92%) identified their specialty as “Other” than Family Medicine or Pediatrics. Most survey participants have been in practice for over 10 years (*n* = 33, 63.46%). All survey participants practice in the United States and most (*n* = 48) practice in the state of Missouri. A summary of the self-reported demographics of survey participants is presented in Table 4.

**Table 4:** Self-reported demographic table for survey participants from section (1) demographics.

Education	*n* (*n*%)
MD	48 (92.31%)
DO	2 (3.85%)
DNP	1 (1.92%)
MSN	1 (1.92%)
PA	0 (0%)
Speciality	
Family medicine	28 (53.85%)
Pediatrics	23 (44.23%)
Other	1 (1.92%)
Subspecialty	
Obesity medicine	1 (1.92%)
Pediatric endocrinology	2 (3.85%)
Developmental pediatrics	1 (1.92%)
Adolescent medicine	1 (1.92%)
Nephrology	1 (1.92%)
Years in practice	
<1 years	0 (0%)
1–3 years	3 (5.77%)
3–5 years	6 (11.54%)
5–10 years	10 (19.23%)
>10 years	33 (63.46%)
Gender	
Cis-man	11 (21.15%)
Cis-woman	38 (73.08%)
Prefer not to answer	3 (5.77%)
RACE (select all that apply)	
Black or AA	2 (3.85%)
American Indian or Alaska Native	3 (5.77%)
Asian	5 (9.62%)
Native Hawaiian or other PI	1 (1.92%)
White	46 (88.46%)
Some other race	2 (3.85%)
Ethnicity	
Not Hispanic or Latino	49 (94.23%)
Hispanic or Latino	2 (3.85%)
Declined to answer	1 (1.92%)
Age	
<20	0 (0%)
20–30	0 (0%)
31–40	21 (40.38%)
41–50	12 (23.08%)
51–60	11 (21.15%)
>60	6 (11.54%)
Declined to answer	2 (3.85%)

### 3.4 User-interface design

Regarding visualizing data, 67% of participants preferred line graphs over bar graphs when visualizing lifestyle data logged by pediatric or adolescent patients (Item 3.2). Additionally, 71% of participants preferred weekly averages to monthly averages when visualizing lifestyle data logged by pediatric or adolescent patients (Item 3.3). Lastly, 65.38% of survey participants (*n* = 34) preferred line graphs with separate health metric (e.g., weight only) data, as opposed to a line graph that combines two health metrics [e.g., weight and blood pressure (BP)].

Section (4) *CommitFit EHR tool,* on the survey, evaluated the visual design of the prototype for Version 3 of the CommitFit EHR tool which was the most updated version at the time the surveys were distributed, as depicted in Figure 2. Respondents rated the statement on a scale of 0–100 with a higher score indicating they agreed significantly with the statement, with 100 being labeled as strongly agree. The highest-rated statement (mean: 78.21/100; std. dev: 20.11) was “*I like the colors of this tool”* (note, the colors refer to the “traffic light” color scheme). The lowest-rated statement (mean: 67.67/100; std. dev: 20.99) was “*I would recommend using this tool to others.”* A complete overview of the *Section* (4) *CommitFit EHR tool* responses is reported in Table 5.

**Table 5:** Overview of the scale responses for survey section four (4) commitFit EHR tool.

Statement	Mean	Std. Dev
I like the layout of this tool.	74.73	18.65
I like the colors of this tool.	78.21	20.11
I like the look and feel of this tool.	72.10	22.23
I anticipate using this tool in the clinic.	67.98	23.65
I would recommend using this tool to others.	67.67	20.99
Information from this tool will help me provide shared decision making with my patients.	76.54	20.42
I can retrieve the information I need easily using this tool.	73.92	21.42
I anticipate that this tool will be useful when I provide health behavior counseling in my clinic.	77.27	18.63
I anticipate that this tool will make providing health behavior counseling in my clinic easier.	75.27	19.07

## 4 Discussion

The EHR and their related visualization tools, such as colors and graphs, have become essential in modern clinical practice (36, 48, 49). The integration of EHR and related tools has the potential to streamline clinical workflow, improve patient outcomes, and increase the efficiency of healthcare systems (50). Additionally, when these EHR tools are co-developed with clinicians, the adaptability and usefulness of these tools is greater (51, 52). Involving users in the design process of EHR tools is more likely to improve the tool's functionality and usability (53). Poor usability of EHRs has been linked to provider dissatisfaction and led to patient safety issues (54).

To comprehensively review the implementation of an EHR tool, developers of EHR tools should first assess users’ workflow and desired tool outcomes, and then reassess if these needs are met after implementation (55). Due to time restraints and demand in clinics, healthcare professionals and providers need streamlined processes (32). EHR tools are much more likely to be adopted if they are easy to use, useful, and easily incorporated in their current workflow. The CommitFit EHR Tool was created with these principles; as a result, survey participants generally approved of the statement “*I anticipate that this tool will make providing health behavior counseling in my clinic easier”* (mean 75.27/100).

Although the scores specific to the utility of the EHR tool were high (“*I anticipate that this tool will be useful when I provide health behavior counseling in my clinic”*, mean 77.27/100), and the majority reported they would use the CommitFit EHR tool (67.98/100) and recommend it to others (67.67/100), these last two statements had the lowest scores. Further evaluation would help elicit why providers liked the look and feel of the CommitFit EHR tool, and why fewer feel that they would use it or recommend it to others. It's possible that they don't see a tool like this fitting into their obesity management workflow, which would explain why the lowest-rated statements were “*I anticipate using this tool in the clinic”* and *“I would recommend using this tool to others.”*

Findings from previous literature align with results from this mixed-method study. We found two qualitative themes (1) clinician participants want the CommitFit EHR tool to facilitate and enhance in-clinic lifestyle counseling with their adolescent patients, and (2) the colors and graphs used in the CommitFit EHR tool should be intentional to improve usability.

(1) Clinician participants want the CommitFit EHR tool to facilitate and enhance in-clinic lifestyle counseling with their adolescent patients.

We designed the CommitFit EHR tool to be paired with the CommitFit mHealth app, to improve lifestyle data accessibility and sharing. Patient-driven data collection from the CommitFit mHealth app may lower the in-clinic data collection burden (56) and will allows clinicians to dedicate more time to patient care (57) with the potential to improve quality of care and patient and provider satisfaction. The CommitFit mHealth app encourages patient engagement (8), which may improve communication and education in-clinic (58). Therefore, the CommitFit EHR tool provides a unique opportunity to facilitate clearer communication and education between clinicians and patients.

Clinician participants need the CommitFit EHR tool to be easy to use and presentable to adolescent patients and their caregivers. The ability to share results from and interact with the CommitFit EHR tool during patient interactions was found to be highly valued by participants in our results. During the first focus group, one participant stated, “*I could see myself turning [the CommitFit EHR tool] around and showing it to the family when they're there for a visit, like to discuss results. So, just having it, at least in a way that appears, you know, a parent and child could interact with the [CommitFit EHR tool] when we're in the office.”* This demonstrates how the CommitFit EHR tool can be used to enhance patient-shared decision making and goal setting. Additionally, the highest-rated survey question “*I anticipate that this tool will be useful when I provide health behavior counseling in my clinic”* (mean 77.27/100) which highlights the CommitFit EHR tool's ability to enhance decision making and usability.

The CommitFit EHR tool dashboard should be simple and designed to interface with patients and their caregivers to enhance these conversations. Information in the EHR should be displayed in a manner that is understandable to both clinicians and patients, which aids in the ease of use of the CommitFit EHR tool. Visualization tools which empower patients and their families to understand their health and lifestyle data can improve patient compliance and engagement (59), leading to better long-term outcomes (60, 61).

As previously noted, impactful visualization tools, such as graphs, can play a pivotal role in improving the effectiveness of clinical workflows, as clinicians face growing demands on their time and expertise (49). Now more than ever, innovative EHR tools like CommitFit offer streamlined and efficient processes. Our qualitative results align well with the previous literature that EHR data visualizations—when properly implemented—allow clinicians to enhance workflow and understanding of complex data and trends (48, 62). Providers are burdened with vast amounts of health data on a daily basis (63–65), and visualization tools help in interpreting this data efficiently, reducing overload and fatigue.

(2) The colors and graphs used in the CommitFit EHR tool should be intentional to improve usability

Based on focus group feedback, the CommitFit EHR tool underwent a color scheme redesign [see Figure 2 (Version 1–4)] with the final tool using blue indicating goal achievement, orange for near-achievement, and pink for non-achievement. It is important to be intentional when choosing EHR color schemes because they can facilitate efficient data interpretation (66), aiding clinicians (48, 67) in informed decision-making for improved patient care (68) quickly and efficiently. Graphs, charts, and an impactful color palette make it easier to identify trends and abnormalities (69–71). With these iterative improvements to color and graphs, survey participants endorsed the statement “*I can retrieve the information I need easily using this tool”* (mean 73.92/100), reflecting the effectiveness of the data visualizations of the CommitFit EHR Tool.

The readability of graphs is a crucial element of effective visualization. Sixty-seven percent (67%) of participants preferred line graphs over bar graphs when visualizing lifestyle data logged by pediatric or adolescent patients (Item 3.2). Line graphs were featured in Version 2 of the CommitFit EHR tool and presented to the second Focus Group; however, during the focus groups after the data was presented as a line graph, participants did not prefer line graphs when applied to the CommitFit EHR tool on the “Patient Selected Goals.” Thus, later iterations of the CommitFit EHR tool feature a bar graph for visualizing patient-reported lifestyle data (via “Patient Selected Goals”) and line graphs for health metrics (i.e., weight or blood pressure percentile).

Both survey and focus group data were carefully considered; however, due to conflicting findings regarding graph displays, focus groups were utilized to explore these differences in preferences. We prioritized the preferences of the focus group over the survey when deciding on colors and the choice between bar and line graphs, as it provided a more nuanced option that appeared more aligned with clinicians’ needs. We hypothesize that the interactive nature of the focus groups enabled a more comprehensive capture of the complexity of clinical needs and considerations. Additional testing of the CommitFit EHR tool in the clinic setting would more definitively answer these questions.

Customizable graphs, enabling users to adjust colors and select between line and bar displays according to their preferences, could offer additional benefits. Moreso, EHR tools should be customizable to protect clinicians from data overload (72–75). We anticipate the CommitFit EHR tool will allow clinicians to customize the graphs and displays for effective decision-making and personalization. Meaningful data presentation and graphs can also aid in early intervention and detection of concerning biometric trends. Issues such as *Y*-axis scale can make a big difference on how data is perceived (76).

Additionally, 71% of participants preferred weekly averages to monthly averages when visualizing graphs of lifestyle data logged by pediatric or adolescent patients (Item 3.3). Thus, both weekly and monthly averages presented in the top right of the lifestyle data graph on the CommitFit EHR tool were included. Users can select whether they view lifestyle data weekly or monthly. The ability of the CommitFit EHR tool to adapt to clinician needs improves ease of use for the primary users of the tool and is anticipated to enhance the clinical workflow (77).

### 4.1 Limitations

Our focus group only had 12 participants with a minimum of six participants per focus group session. A limitation of this study was the focus group distribution and sample size, with no representation from non-physician providers. Thus, there is a potential bias toward physician use, which does not consider the normal distribution of healthcare providers. Due to the small sample size and small focus groups, it is difficult to validate our qualitative results. However, by the end of the third focus group we had reached a saturation of ideas, which limited the benefit of conducting additional focus groups (31). Saturation was determined to be reached when limited to no novel feedback was provided during the focus groups. Additionally, our survey had a relatively small sample size (*n* = 52). The scaled questions had high standard deviations, showing variability in the opinions on the CommitFit EHR tool during Version 3 of the prototype and a small sample size. We did not evaluate user analytics or EHR tool effectiveness in clinical practice. In the future, more research is needed with a larger and more diverse sample and to explore clinician user analytics for the CommitFit EHR tool to evaluate real world use. Furthermore, the effectiveness of the CommitFit mHealth and CommitFit EHR tool as a weight management intervention needs to be evaluated.

## 5 Conclusion

Childhood and adolescent obesity continue to be a prevalent public health concern. Thus, there is a growing need for novel weight management interventions, including EHR tools, supporting clinicians working to reduce pediatric obesity. We created the CommitFit EHR tool to pair with the CommitFit mHealth app for adolescent weight management and to provide positive health behaviors. We used a mixed-methods approach to identify the provider functionality and design needs, and preferences when developing the CommitFit EHR tool. Two qualitative themes were (1) Participants want the CommitFit EHR tool to facilitate and enhance in-clinic lifestyle counseling with their adolescent patients, and (2) the colors and graphs used in the CommitFit EHR tool should be intentional to improve usability. The layout of the CommitFit EHR tool, including the presentation of graphs, was crucial to improve workflow and functionality. During each step of feedback from focus group sessions and the survey, the design of the CommitFit EHR tool was updated in a stepwise fashion. This iterative process allowed the CommitFit EHR tool to be co-developed by clinician users with a user-centered approach. As a result of being co-developed by clinician users, the CommitFit EHR tool may be more responsive as an intervention in the clinic. More research is needed to explore clinician user analytics for the CommitFit EHR tool to evaluate real-time workflow needs. Lastly, CommitFit mHealth and EHR tool as a weight management intervention needs to be evaluated to determine its effectiveness in facilitating patient health behavior change and preventing or managing obesity.

## Data Availability

The raw data supporting the conclusions of this article will be made available by the authors, without undue reservation.
